# Vascular ring: prenatal diagnosis and prognostic management based on sequential cross-sectional scanning by ultrasound

**DOI:** 10.1186/s12884-023-05637-y

**Published:** 2023-05-02

**Authors:** Yi Zhou, Yuanyuan Zhou, Tingting Yu, Wanyan Li, Jingshu Zhang, Chaoxue Zhang

**Affiliations:** Department of Ultrasound, The First Affiliated Hospital of Anhui Medical University, 218 Jixi Road, Hefei, Anhui 230022 China

**Keywords:** Vascular ring, Prenatal diagnosis, Prognostic management, Dynamic sequential cross-sectional

## Abstract

**Background:**

In terms of embryonic origin, vascular ring is a congenital anomaly in which the aortic arch and its branches completely or incompletely encircle and compress the trachea or esophagus. Early and accurate diagnosis of a vascular ring is the key to treatment. Prenatal diagnosis mainly relies on fetal echocardiography, but the rate of missed diagnosis and misdiagnosis is still very high, and the prognosis has not been evaluated. The aim of this study was to investigate the accuracy of prenatal diagnosis and to evaluate the prognosis semi-quantitatively according to the shape of the ring and the distance between the vessel and the trachea.

**Methods:**

From 2019 to 2021, 37,875 fetuses underwent prenatal ultrasound examination in our center. All fetal cardiac examinations were performed using the fetal echocardiography method proposed by the American Institute of Ultrasound in Medicine (AIUM) combined with dynamic sequential cross-sectional observation (SCS). For SCS, the standard abdominal section was taken as the initial section, and the probe was moved cephalically along the long axis of the body until the superior mediastinum had disappeared. If a vascular ring was found, the shape of the ring and the distance of the branch to the airway were observed. The distance relationship with the airway was divided into three grades: I-III; the closer the distance, the lower the grade. The vascular rings were monitored every 4 weeks before birth. All were monitored before surgery or 1 year after birth.

**Results:**

A total of 418 cases of vascular rings were detected. There was no missed diagnoses or misdiagnoses by SCS. The vessels formed different shaped rings according to their origin and route. Grade I, “入” and “O” rings have a poor prognosis and are associated with the highest risk of respiratory symptoms.

**Conclusions:**

SCS can accurately diagnose vascular rings before delivery, evaluate the shape and size of the rings to conduct prenatal monitoring of children until birth, which plays a guiding role in airway compression after birth.

## Background

In terms of embryonic origin, vascular ring is a congenital anomaly in which the aortic arch and its branches completely or incompletely encircle and compress the trachea or esophagus [1]. The common vascular rings are left aortic arch with right subclavian artery vagus (LAA with ARSA), right aortic arch with mirror-image branching (RAA with mirror-image branching), right aortic arch with left subclavian artery vagus (RAA with ALSA), double aortic arch (DAA), and pulmonary artery sling (PAS). The different types of vascular rings have different incidences, clinical manifestations and severities. Symptoms frequently encountered include noisy breathing and a barky cough, recurrent upper respiratory infections, wheezing, exertional dyspnea, and dysphagia. Children often present with symptoms in the first few months of life and require surgery within the first year of life [2, 3]. Delaying treatment may result in sudden death or residual tracheobronchial damage [4]. Postpartum diagnosis mainly depends on MDCT [5]. Prenatal diagnosis of vascular ring is relatively difficult. In recent years, the value of the three-vessel tracheal view in fetal echocardiography in the diagnosis of vascular ring diseases has been recognized [6–8], but the guidelines state that only fetuses with high risk factors receive specialized fetal echocardiography. Most fetuses receive only a basic cardiac examination, and the success of detection depends on the operator’s experience and knowledge of the disease [9]. Therefore, the rate of missed diagnoses and misdiagnoses remains high, in particular, for isolated vascular rings, prenatal data statistics are only 1 in 1000 [6]. There is also a lack of quantitative assessment of the risk of fetal airway compression caused by the vascular ring [10]. The purpose of this study was to investigate the value of prenatal ultrasound in the integrated management of vascular ring.

## Method

From January 2019 to December 2021, 37,875 fetuses underwent systematic prenatal ultrasound examination in our center. The gestational age was 18–33 weeks, and the pregnant women were between 20 and 48 years old. All fetuses have their systems checked in detail according to the guidelines, and if abnormalities were found, their family history was registered, with invasive chromosomal tests performed if necessary. All fetuses examined at our center were followed up after birth or after induced labor. All pregnant women provided written informed consent for fetal examination, and the study was approved by the First Affiliated Hospital of Anhui Medical University’s Institutional Review Board (PJ2022-08-46). Equipment: Voluson: E8/10 Expert systems (General Electric Medical Systems Kretztechnik).

### Scanning method

All fetal cardiac examinations were performed using the fetal echocardiography method proposed by the American Institute of Ultrasound in Medicine (AIUM), including the following: abdominal view, four-chamber view, left ventricular outflow tract, right ventricular outflow tract, pulmonary artery bifurcation with branches, three-vessel view, short-axis views (“low” for ventricles and “high” for outflow tracts), and long-axis view (aortic arch, ductal arch, superior and inferior venae cavae) [9]. The running and spatial relationship of each vascular structure were observed by continuous and dynamic sequential cross-sectional observation (SCS) [11]. If a vascular ring was found, the shape of the ring, abnormal vascular running and structural space relationship were observed through SCS. The vertical distance from the vessels forming the vascular ring to the outermost layer of the vascular wall and the outermost layer of the trachea wall were measured and graded. The distance between the vascular ring and trachea was evaluated by observing whether there were branch vessels in the vascular ring and their running pattern. Airway compression by the vascular ring was evaluated at the nearest distance level. The vascular rings were monitored every 4 weeks before birth. All were monitored before surgery or 1 year after birth, but they were not monitored during labor induction.

### SCS

**Fig. 1:**
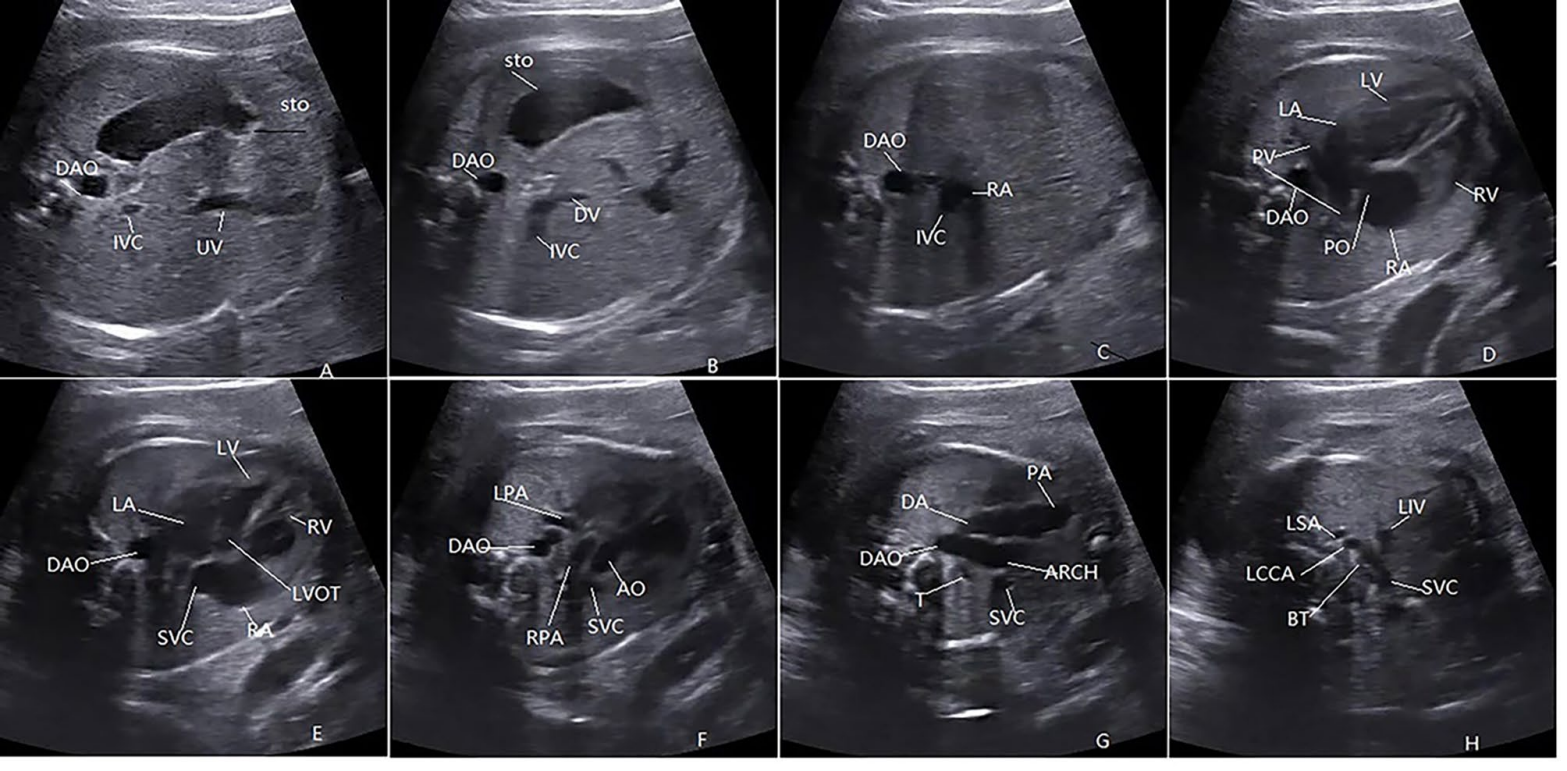
Section requirements of SCS scanning method:the upper-abdomen view [6]: (**A**) Showing the fetal STO, cross-section of the abdominal DAO, IVC and UV. (**B**) The slightly more cephalad view, showing STO, DAO, IVC and DV. (**C**) The slightly more cephalad view, showing DAO, IVC drain in the RA. (**D**) The four-chamber view, showing the RV, LV, RA, LA, FO, PV, DAO. (**E**) The five-chamber view, showing the LVOT, SVC and DAO. (**F**) The slightly more cephalad view revealing the bifurcation of the pulmonary arteries, showing the LPA, RPA, SVC and cross-sections of the DAO and AO. (**G**) The three vessel and trachea 3VT view, showing the PA, DA, SVC, DAO, AA and T. (**H**) The most cephalad transverse view showing SVC, LIV, three branches of the aortic arch, BT, LCCA, LSA, respectively. The copyright of the FIG has been granted by the author

Taking the standard abdominal section as the initial section, the probe was moved cephalically along the long axis of the body until the superior mediastinum had disappeared. During the scanning process, the probe was moved appropriately according to the continuity of the cross-section and image quality of the structure to be observed (Fig. 1).

### Airway assessment grade

In the enlarged image, the target area was zoomed into, which would occupy 1/3 of the screen, I:1) The tissue structure between the trachea and vascular ring then disappeared. 2) During the SCS scan, the inner diameter and shape of the trachea in the vascular ring changed or did not change. II:1) A small amount of tissue structure can be seen between the vascular ring and the trachea. 2) The vertical distance between the ring and the outer wall of the trachea can be measured by image magnification. III:1) The tissue structure can be seen between the vascular ring and the trachea. 2) The vertical distance between the vascular ring and the lateral wall of the trachea can be easily measured.

### Statistical analysis

Statistical software SPSS 23.0 was used to process the data. The Kruskal‒Wallis test was used to conduct the nonparametric test of multiple independent samples, and the single-factor ANOVA test was used to compare the differences between the two independent samples. P < 0.05 was considered statistically significant.

### Result: 1

**Fig. 2:**
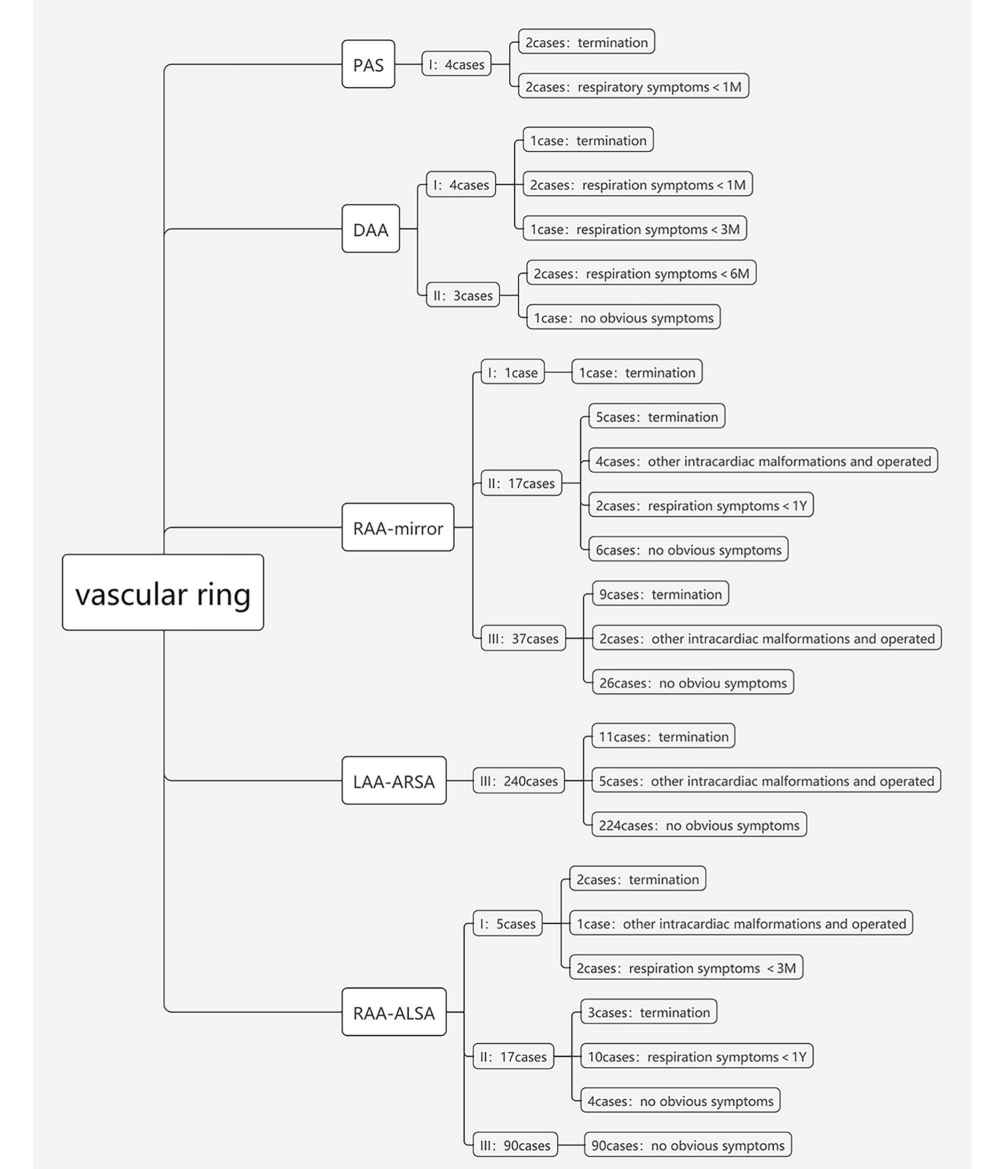
Prenatal diagnosis vascular ring, airway assessment and prognosis

**Fig. 3:**
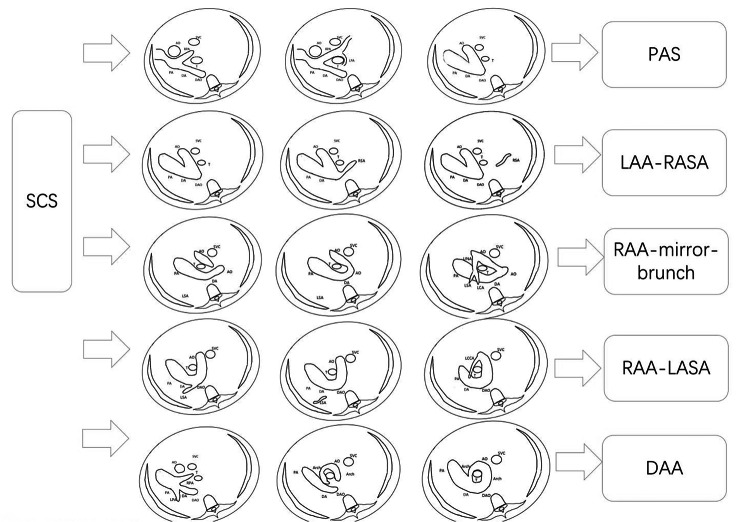
Summary of experience in diagnosis of fetal vascular rings by SCS scaning

A total of 418 (418/37,875) cases of vascular rings were detected by ultrasound, with a gestational age between 18 and 33 weeks, and these included 240 cases of LAA with ARSA, 55 cases of RAA with Mirror-Image Branching, 112 cases of RAA with ALSA, 7 cases of DAA (4 cases DAA with symmetric arches, 3 cases DAA with hypoplastic left arch), and 4 cases of PAS. There were 23 cases of LAA with ARSA, and 2 cases of pulmonary artery sling in the middle of pregnancy were not found to be abnormal in other hospitals. In our hospital, the diagnosis was made at 32 weeks of late pregnancy. Three fetuses were referred to our hospital with a double aortic arch, which indicated a right-sided aortic arch with a mirroring branch. A total of 1196 of 37,875 fetuses were not followed up, and the rest were followed up after birth or induced labor. There was no obvious missed diagnoses or misdiagnoses of vascular ring disease (Table 1; Fig. 2). Based on the ultrasonographic characteristics for diagnosing vascular rings in a continuous cross-section sequence, we summarized the diagnostic map of this disease (Fig. 3).

**Table 1:** Prenatal diagnosis of congenital vascular ring

Types of vascular rings	The number of cases	Accompanied by intracardiac malformation	Accompanied by extracardiac malformation	Invasive chromosome testing
LAA-ARSA	240	ventricular septal defect (4) pulmonary stenosis (1) persistent left superior vena cava (2)	fetal growth restriction (3) strephenopodia (4) cleft lip (3) nasal bone dysplasia (3) abnormal urinary system (3) ascites (2) hydrothorax (3) embryo lymph cyst (1) dacryocystocele (2) ventricular dilatation (3) dysgenesis of corpus callosum (2) choroidplexuscyst(3)	Trisomy 18 (2) Trisomy 21 (2) 22q11.21 (1)
RAA-MIRROR-IMAGE BRANCHING	55	ventricular septal defect (5) persistent left superior vena cava (4) Tetralogy of fallot (2) Persistent truncus arteriosus (2) Transposition of the great vessels (3) Double outlet right ventricle (2) pulmonary stenosis (2) Hydropericardium (2) Tricuspid regurgitation (2)	cleft lip (2) Butterfly vertrbra (1) strephenopodia (3) Fingers overlapping (1) ventricular dilatation (3) Choroidplexuscyst (2) abnormal urinary system (3) ascites (1) nasal bone dysplasia (2) ventricular dilatation (2) abnormal urinary system (1)	Trisomy 18 (2) Trisomy 21 (1) 22q11.21 (1)
RAA-ALSA	112	ventricular septal defect (1) persistent left superior vena cava (1)	cleft lip (1) Adactylous (3) Genital abnormalities (1) strephenopodia (2) Ependymal cyst (3)	Trisomy 21 (1) 22q11.21 (1)
DAA	7	0	Choroidplexuscyst (1)	22q11.21 (1)
PULMONARY ARTERY SLING	4	0	strephenopodia (1) dysgenesis of corpus callosum (1) ventricular dilatation (2) cleft lip (1)	Trisomy 18 (1)

Two cases of PAS were associated with other extracardiac malformations, and 1 case of DAA with symmetric arches was associated with other extracardiac malformations. There were 14 patients with other extracardiac malformations and 22 patients with intracardiac malformations in the RAA with mirror-image branching. 27 cases were associated with other extracardiac malformations, and 7 cases were associated with other intracardiac malformations in the LAA-ARSA. 9 cases of RAA-ALSA were associated with other extracardiac malformations, and 2 cases were associated with intracardiac malformations. A total of 245 of the 418 fetuses underwent chromosomal testing, 13 of whom had chromosomal abnormalities (Table 1).

**Fig. 4:**
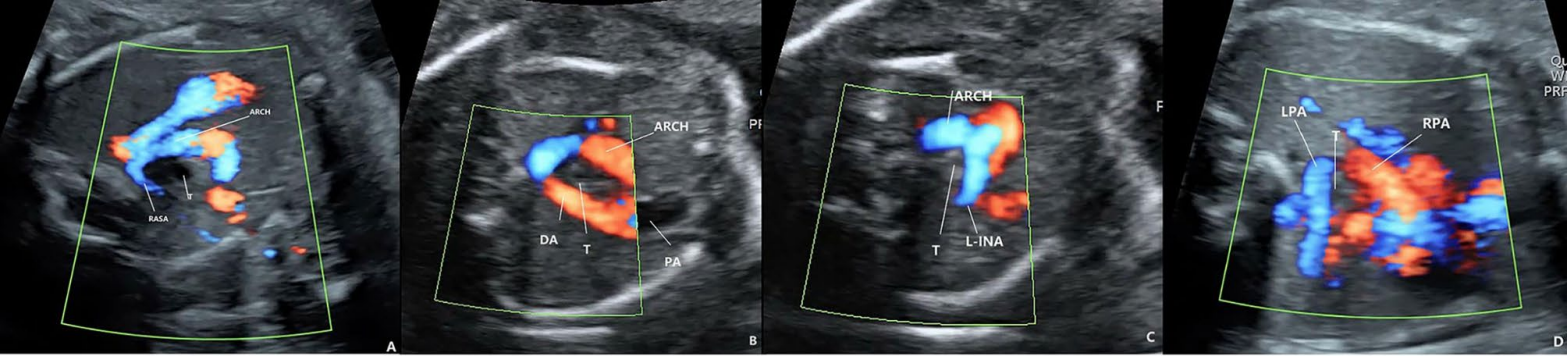
The distance between the ring and the airway **A**: Grade III: “U”, the tissue between the trachea and the blood vessels forming the ring is clearly visible and far away **B**: Grade II: “U”, the tissue between the trachea and the blood vessels forming the ring is visible, and the distance is slightly close, so direct measurement is difficult **C**: Grade I: “U”, the tissue between the trachea and the blood vessel on the side of the ring is gone **D**: Grade I: PAS, “入”, the tissue between the trachea and the blood vessel on the side of the ring is gone, tracheal cross section deformation

The grade of final airway distance and shape of each blood vessel ring before birth were as follows: PAS: “入” type ring, all 4 cases were classified as grade (I) DAA: “O” type ring, 4 cases of grade I and 3 cases of grade (II) RAA mirror-Imaging Branching: “U” type ring, the L-INA is seen in the vascular ring ascending aorta to the left shoulder. 1 case was grade I, 17 cases were grade II, and 37 cases were grade (III) LAA-ARSA: “C” type ring, 240 cases of grade III. RAA-ALSA: “U” type ring, the left common carotid artery is seen in the ring running to the left neck. Five cases were grade I, 17 cases were grade II, and 90 cases were grade III. These data are from the perspective of prognosis and did not include those whose family members actively chose to undergo surgery or terminate pregnancy. (Table 2; Fig. 4)

**Table 2:** Shape of the vascular ring and its distance to the trachea and esophagus

Types of vascular rings	The shape of the vascular ring	The composition of the vascular ring	Its distance to the trachea and esophagus(last check-up before birth),
LAA-ARSA	C	ARSA,LAA	III(240cases)
RAA-MIRROR-IMAGE BRANCHING	U	RAA,LDA	I(1case),II(17cases),III(37cases)
RAA-ALSA	U	RAA,LDA	I(5cases),II(17cases),III(90cases)
DAA	0	R-ARCH,L-ARCH	I(4cases),II(3cases)
PULMONARY ARTERY SLING	入	LPA-Sling	I(4cases)

**4.** The statistical results showed that the outcomes of the children with different shapes of vascular rings were significantly different (P < 0.001). According to the rank mean, the observation results of the C-type patients were the best, while those of the 入-type and O-type patients were poor. Single-factor ANOVA showed that the difference between the two groups was statistically significant (P < 0.001) (Tables 3 and 4). For different distance grading groups, the higher the distance grading of patients, the better the observation results, and the difference was significant (P < 0.001) (Tables 5 and 6).

**Table 3:** Kruskal-Wallis test based on prognosis of ring shape

The shape of the vascular ring	Number of Samples	Rank mean	Kruskal-Wallis
“入”	2	2.50	X^2^ = 126.583 P = 0.000(df = 3)
“O”	6	37.50	
“U”	140	178.71	
“C”	224	197.00	

**Table 4:** Single factor ANOVA test of prognosis based on ring shape:

The shape of the vascular ring		ANOVA
“入”	“O”	P = 0.000
	“U”	P = 0.000
	“C”	P = 0.000
“O”	“U”	P = 0.000
	“C”	P = 0.000
“U”	“C”	P = 0.002

**Table 5:** Kruskal-Wallis test based on prognosis of distance grading :

Distance grading	Number of Samples	Rank mean	Kruskal-Wallis
Grade I	7	4.00	X^2^ = 260.006 P = 0.000(df = 2)
Grade II	25	94.80	
Grade III	340	197.00	

**Table 6:** Single factor ANOVA test of prognosis based on ring shape:

Distance grading		ANOVA
Grade I	Grade II	P = 0.000
	Grade III	P = 0.000
Grade II	Grade III	P = 0.000

## Discussion

The vascular ring is the annular or semiannular structure surrounding the trachea and esophagus that can compress the trachea or esophagus due to abnormal development of the primitive arterial system in the embryo [12]. In more severe cases, symptoms of compression may occur during the fetal period or after birth. The earlier the diagnosis of vascular ring disease and the timely surgical treatment of patients with possible compression, the better the prognosis [13]. With the rich experience of prenatal diagnosis, the use of three-vessel tracheotomy for the diagnosis of vascular ring diseases has been widely recognized in recent years [14]. It has been reported that the prenatal detection rate of isolated vascular rings has increased from 2.4 to 10,000 to 7 per 10,000 [8]. But there are still misdiagnoses and missed diagnoses of vascular rings. The reasons may be as follows: (1) Most fetuses receive only a basic cardiac examination, only high-risk fetuses underwent fetal echocardiography, and the success of detection depends on the operator’s experience and knowledge of the disease. (2) The examination method of fetal echocardiography is mainly the nine-section method, and the aortic arch and above vascular branches are mainly dependent on the observation of the long axis. With the increase in gestational age and fixed fetal position, the display rate of the long axis section is decreased [9, 15]. It is even more difficult to track the course of blood vessels from the long axis and assess their relationship to the surrounding structures. In this study, 23 cases of LAA-ARSA and 2 cases of PAS were missed in the second trimester. We analyzed the characteristics of the sonogram because the origin and movement of the blood vessels were not well observed. Three cases of RAA mirror-branch imaging were misdiagnosed as DAA and were identified as O-ring simply from the shape of the ring, which was actually the overlap of the vascular section, and the mirror branch did not merge into the descending aorta behind the trachea. This study presents a method of scanning the fetal heart with continuous cross-sectional sequence scanning. The operator simply moves from a standard section of the abdominal circumference along the long axis of the body to the cephalic side. Following the cross-section of the great vessels of the heart until the mediastinum disappears, the method does not include the long axis section of the heart, which is less affected, convenient and easy to master, and easy to understand by tracing the origin of abnormal blood vessels through a continuous cross Sect.  [11]. We attempt to summarize the diagnostic ideas of SCS in the diagnosis of common vascular ring diseases, which is more conducive to the diagnosis and differential diagnosis of non-professional echocardiologists. In addition to the diagnosis of common vascular ring, in the past two years, the definition of vascular ring has been expanded, and other rare vascular abnormalities that constitute a ring structure are also included in the vascular ring, such as innominate artery compression syndrome, right aortic arch with right ductus arch. And the abnormal course of blood vessels that do not form a ring structure but may cause tracheal compression, such as left brachiocephalic vein abnormalities. Prenatal diagnosis is often neglected due to the rarity of the disease and its prognosis. In theory, the SCS method based on CT images can continuously track the course of abnormal blood vessels and the spatial relationship with surrounding structures through the transverse section, which can be easier to diagnose. In the future work, we will verify such rare abnormal vascular malformation diseases. [11, 16–18].

Early diagnosis of congenital vascular rings is intended to improve the symptoms of compression in patients with airway compression [19]. With the increase of prenatal detection rate of vascular rings, the incidence of rare diseases has been realized to be twice that of tetralogy of Fallot [8], and the evaluation of the relationship between vascular rings and airways is more important. However, there are few articles on the prenatal diagnosis of vascular ring diseases and with qualitative or quantitative assessments of airway prognosis in PubMed [10]. Several cases of vascular rings suggested by prenatal diagnosis and requiring tracheal intubation immediately after birth were reported, and no airway condition was suggested prenatally [20, 21]. Achiron et al. first proposed prenatal assessment of vascular rings and airway compression, but the study limitations were subjective qualitative rather than quantitative assessment of airway compression [10]. Chest X-ray is not routinely recommended to diagnose vascular rings after birth, even though it can be useful to show the location of the aortic arch in relation to the trachea [22]. Computed tomography with angiography (CTA) is an important tool that allows for a careful simultaneous assessment of vascular abnormalities and airway involvement [23]. To assess the prognosis of congenital vascular ring disease in the fetus and by following the principle of CT in postnatal diagnostic evaluation, we proposed the dynamic evaluation of the influence of vascular rings on the airway by continuous transverse scanning during the fetal period. The distance between the airway and the blood vessels forming the ring was managed by semi-quantitative grading. This set of data shows the following: (1) Compared with the other types of vascular rings, PAS may have compression symptoms in the fetal period, and the prognosis is relatively poor. If it can be maintained until birth, immediate surgery is needed to improve the symptoms, and the improvement of symptoms after surgery is still affected by tracheomalacia. (2) The second most common type to have a poor prognosis is DAA. In these data, in addition to terminating fetuses, 5 babies developed respiratory symptoms in the first six months of life, which is mainly related to the “O” ring and closed ring formed by double aortic arches. (3) RAA mirror-brunching imaging and RAA-ALSA can also be involved. The “U”-shaped ring formed by the right aortic arch and ductal arch has a relatively low risk of airway compression. However, there are branches in the vascular ring, and when the branches are close to the airway, they and the right arch may bring some risks to airway compression. According to this set of data, the ICCA branch of RAA-ALSA may present a higher risk of airway compression than the L-INA of RAA mirror-brunching imaging, but the risk is lower than that of the first two vascular rings. The prognosis is relatively good. (4) The farthest distance was LAA-ARSA, and there was no obvious airway compression in the group with the best prognosis. These results are consistent with literature reports. ARSA does not demonstrate any apparent clinical symptoms, but it is used as a soft marker of ultrasound. When ARSA is observed with other ultrasound abnormalities, the risk of pathogenic CNV is increased remarkably; therefore, a prenatal diagnosis is very important [24].

Based on this single-center sample, we believe that the shape of the vascular ring, the route of the branching vessels, and the distance from the airway are critical to the prognosis of these diseases. Dynamic sequential cross-sectional scanning can observe these aspects very well and intuitively, has more advantages than the long axis, and can carry out prenatal monitoring and management of the fetus until birth.

The shortcomings of this group study are as follows: (1) Due to the single-center patients collected and followed up in this cohort, the incidence of some types of vascular ring diseases was relatively low, and the number of positive samples was relatively small because the patients rarely contained vascular rings. Also, our research will not stop. If we encounter vascular rings, which are rare, we will continue to verify the feasibility of our method. (2) For positive cases, the patients were followed up for only one year after birth, and the families of some patients received active surgical treatment, so long-term evaluation could not be achieved. (3) There is a lack of a quantitative cutoff value for the safe distance from the vascular ring to the airway. At present, we are still collecting much data and following up on the prognosis, trying to analyze and find the safe distance between the vascular ring and the trachea and providing guidance for real clinical treatment evaluation.

## Conclusion

SCS can accurately diagnose congenital vascular rings before delivery and can evaluate the shape and size of the rings well to conduct prenatal monitoring of children until birth, which plays a guiding role in airway compression after birth.

## Data Availability

The datasets used and/or analyzed during the current study are available from the corresponding author on reasonable request.

## Abbreviations

LAA with ARSA: Left aortic arch with right subclavian artery vagus
RAA with Mirror-Image Branching: Right aortic arch with mirror-image branching
CTA: Computed tomography with angiography
RAA With ALSA: Right aortic arch with left subclavian artery vagus
DAA: Double aortic arch
PAS: Pulmonary Artery Sling
SCS: Sequential Cross-Sectional observation
STO: Stomach
DAO: Descending aorta
IVC: Inferior vena cava
UV: Umbilical vein
DV: Ductus venosus
HV: Hepatic vein
LV: Left ventricular
LA: Left atrium
RV: Right ventricular
RA: Right atrium
FO: Foramen ovale
PV: Pulmonary vein
LVOT: Left ventricular outflow tract
SVC: Superior vena cava
LPA: Left pulmonary artery
RPA: Right pulmonary artery
PA: Pulmonary artery
AO: Aorta
DA: Arterial duct
ARCH: Aortic arch
T: Trachea
LIV: Left innominate vein
LSA: Left subclavian artery
LCCA: Left common carotid artery
BT: Brachiocephalic trunk
